# Association between vasoactive–inotropic score, morbidity and mortality after heart transplantation

**DOI:** 10.1093/icvts/ivad055

**Published:** 2023-04-17

**Authors:** Joanna Tohme, Mickael Lescroart, Jérémie Guillemin, Pascal Orer, Pauline Dureau, Shaida Varnous, Pascal Leprince, Guillaume Coutance, Adrien Bouglé

**Affiliations:** Department of Anesthesiology and Critical Care Medicine, Cardiology Institute, Sorbonne University, GRC29, AP-HP, Pitié-Salpêtrière Hospital, Paris, France; Department of Thoracic and Cardiovascular Surgery, Cardiology Institute, Sorbonne University, AP-HP, Pitié-Salpêtrière Hospital, Paris, France; Department of Anesthesiology and Critical Care Medicine, Cardiology Institute, Sorbonne University, GRC29, AP-HP, Pitié-Salpêtrière Hospital, Paris, France; Department of Anesthesiology and Critical Care Medicine, Cardiology Institute, Sorbonne University, GRC29, AP-HP, Pitié-Salpêtrière Hospital, Paris, France; Department of Anesthesiology and Critical Care Medicine, Cardiology Institute, Sorbonne University, GRC29, AP-HP, Pitié-Salpêtrière Hospital, Paris, France; Department of Thoracic and Cardiovascular Surgery, Cardiology Institute, Sorbonne University, AP-HP, Pitié-Salpêtrière Hospital, Paris, France; Department of Thoracic and Cardiovascular Surgery, Cardiology Institute, Sorbonne University, AP-HP, Pitié-Salpêtrière Hospital, Paris, France; Department of Thoracic and Cardiovascular Surgery, Cardiology Institute, Sorbonne University, AP-HP, Pitié-Salpêtrière Hospital, Paris, France; Department of Anesthesiology and Critical Care Medicine, Cardiology Institute, Sorbonne University, GRC29, AP-HP, Pitié-Salpêtrière Hospital, Paris, France

**Keywords:** Heart transplantation, Vasoactive–inotropic score, Primary graft dysfunction, Prognostic score, Mortality

## Abstract

**OBJECTIVES:**

The aim of this study was to evaluate the association between vasoactive–inotropic score (VIS), calculated in the 24 h after heart transplantation, and post-transplant mortality and morbidity.

**METHODS:**

This was an observational single-centre retrospective study. Patients admitted to surgical intensive care unit after transplantation, between January 2015 and December 2018, were reviewed consecutively. VIS_max_ was calculated as dopamine+ dobutamine+ 100 × epinephrine + 100 × norepinephrine + 50 × levosimendan + 10 × milrinone (all in µg/kg/min) + 10 000 × vasopressin (units/kg/min), using the maximum dosing rates of vasoactive and inotropic medications in the 24 h after intensive care unit admission. The primary outcome was mortality at 1 year post-transplant. The secondary outcomes included length of stay, duration of mechanical ventilation and inotropic support and the occurrence of septic shock, ventilator-associated pneumonia, bloodstream infection or renal replacement therapy.

**RESULTS:**

A total of 151 patients underwent heart transplantation and admitted to intensive care unit. The median VIS_max_ was 39.2 (interquartile range = 19.4–83.0). VIS_max_ was independently associated with 1-year post-transplant mortality, as well as recipient age [hazard ratio (HR) = 1.004, *P*-value = 0.013], recipient gender (female to male: hazard ratio = 2.23, *P*-value = 0.047) and combined transplantation (hazard ratio = 2.85, *P*-value = 0.048). There was a significant association between VIS_max_ and duration of mechanical ventilation (*P*-value < 0.001), length of stay (*P*-value = 0.002), duration of infused inotropes (*P*-value < 0.001), occurrence of bloodstream infections, septic shocks, ventilation-acquired pneumonia and renal replacement therapy.

**CONCLUSIONS:**

VIS_max_ calculated during the first 24 h after postoperative intensive care unit admission in transplanted patients is independently associated with 1-year mortality. In addition, length of stay, duration of mechanical ventilation and infused inotropes increased with increasing VIS_max_.

**Figure:**
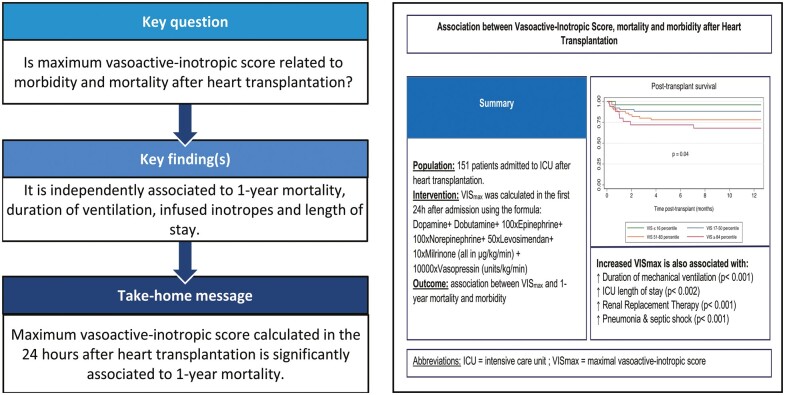

## INTRODUCTION

Despite major advances in durable mechanical circulatory support (MCS), heart transplantation (HTx) remains the most valuable therapeutic option for patients with end-stage heart failure [1, 2]. More than 6000 HTx procedures are performed each year worldwide, with a median post-transplant survival >12 years [3–5]. Primary graft dysfunction (PGD) is common complication after HTx and represents the leading cause of early post-transplant mortality [6, 7]. International guidelines introduced the first consensual definition of the severity of PGD using a four-level scale: mild, moderate, severe PGD-left ventricle (PGD-LV) and PGD-right ventricle [8]. In this grading scale, inotropic/vasopressors requirements were summarized as a dichotomous variable with a threshold defined arbitrary, thereby neglecting a part of the spectrum of inotropic requirements after HTx. Whether the detailed evaluation of inotropic requirements is independently associated with post-transplant outcomes on top of the current PGD classification remains unclear. The vasoactive–inotropic score (VIS) is a score summarizing the level of inotropic/vasopressors requirements calculated according to the type and doses of drugs [9]. It is usually calculated as a mean of inotropic support during a pre-specified period. This score has been associated with prognosis in multiple situations, including cardiac surgery [10–13], but data are scarce after HTx [14, 15]. Recently, VIS has been shown to be associated with poor short-term outcomes after paediatric HTx, but not independently associated with mortality [14]. The VIS has recently been updated as VIS_max_ (defined by the maximum doses of inotropes/vasopressors received during the first 24 h). VIS_max_ appeared to be a more valuable parameter after cardiac surgery [13, 16], but has not yet been evaluated in adult patients undergoing HTx. We aimed to determine whether postoperative VIS_max_ (i) was independently associated with post-transplant mortality and morbidity and (ii) could refine the prognostic value of the current international PGD classification.

## METHODS

### Ethical statement

This study was approved by the ethics committee for research of French Society of Anesthesia and Intensive Care Medicine on May 4, 2021 (IRB 00010254-2021-095). Due to the retrospective non-interventional design of the study and in line with French regulation policy, signed informed consent was waived. The study was conducted in accordance with Declaration of Helsinki principles.

### Study design and population

We conducted an observational single-centre retrospective study in the Surgical ICU of Cardiology Institute at La Pitié-Salpêtrière University Hospital (Paris–France). Patients admitted to surgical ICU after HTx between January 2015 and December 2018 were included. Data were anonymously collected using computerized medical records.

### Outcomes measures

The primary outcome was all-cause mortality at 1-year post-transplant. Secondary outcomes included number of days free from mechanical ventilation and inotropic support during the first 28 days post-HTx, and the occurrence of bloodstream infection, septic shock, ventilator-associated pneumonia (VAP) or renal replacement therapy (RRT).

### Vasoactive–inotropic score

VIS was calculated using the formula proposed by Yamazaki *et al.* [12]: VIS = dopamine + dobutamine + 100 × epinephrine + 100 × norepinephrine + 50 × levosimendan + 10 × milrinone (all in µg/kg^/^min) + 10 000 × vasopressin (units/kg/min). VIS_max_ was calculated using the maximum dosing rates of vasoactive and inotropic medications during the first 24 h after ICU admission. Study population was stratified by VIS_max_ percentiles (<16th, 17th–50th, 51th–83th and >84th percentile).

### Treatment protocols

Heart implantation was performed orthotopically using bicaval technique. The graft ischaemic time was defined as the time interval between the application of aortic cross-clamp in the donor and removal of aortic cross-clamp in the recipient. Celsior solution was used in donor heart preservation.

Immunosuppression after HTx was administered based on a standard protocol described previously [17]. PGD is defined as severe ventricular dysfunction of the donor graft which fails to meet the circulatory requirements of the recipient in the immediate post-transplant period. It differs from secondary graft dysfunction, which is when a discernible cause for allograft dysfunction is identified. The monitoring and management of PGD is detailed in Supplementary Material, Methods.

### Database and data collection

Baseline clinical data on donors and recipients from Paris were obtained from the national registry CRISTAL (Agence de la Biomédecine, French National Agency for Organ Procurement). Anonymized data from this registry are prospectively entered by dedicated staff at specific time points for each patient (day of listing, day of transplant) and are updated annually thereafter. This database is regularly audited. The extraction was carried out on 1 November 2021. We collected detailed characteristics of recipients and their donors including preoperative variables, postoperative variables in the ICU, postoperative variables during the first-year post-transplant (Supplementary Material, Methods).

### Data availability statement

In accordance with the regulations, the data are stored locally in a secure database and are accessible in case of need to provide more details.

### Statistical analysis

Categorical variables were described by frequencies and compared by chi-squared tests. Continuous variables were described by their means (± standard deviation) or medians [± interquartile range (IQR)] and compared Kruskal–Wallis non-parametric test. Cumulative survival curves for the time-to-event analyses were constructed according to the Kaplan–Meier method and compared with the log rank test.

#### Primary outcome

Cox regression was used to evaluate the association between VIS_max_ groups (<16th, 17th–50th, 51th–83th and >84th percentile) and all-cause mortality during the first-year post-transplant. Candidate factors were selected when univariate likelihood ratio test *P*-value was <10%. Descending selection was then used and the final multivariable model selected factor significant at 5%. The additional prognosis value of VIS_max_ on top of current PGD classification was assessed by analysing discrimination (comparison of concordance statistic) and reclassification indices [net reclassification improvement (NRI) and integrated discrimination improvement (IDI)]. The discrimination of the models was evaluated with the concordance statistic and were compared using the STATA’s ‘somersd’ package. To evaluate the robustness of the association between VIS_max_ and mortality, 200 bootstrap samples were generated (random sample with replacement, stratified by type of circulatory support at transplant); in each sample, a multivariable Cox model was generated using the same methodological approach described earlier.

#### Secondary outcomes

The association between VIS_max_ and (i) binary outcomes (septic shock, VAP, bloodstream infection or RRT) was analysed using logistic regression and (ii) the number of days alive free from ventilation or inotropes during the first 30 days post-HTx by comparing and analysing the trend of number of days across VIS_max_ categories using the non-parametric Kruskal–Wallis and the Cuzick test for trend, respectively.

A *P*-value <0.05 was considered statistically significant. Statistical analyses were performed with STATA/SE 14.2 (StataCorp, Texas, USA).

## RESULTS

### Study population

Between January 2015 and December 2018, 151 patients were transplanted and admitted to surgical ICU. Demographic and clinical characteristics of the population are described in Table 1. Mean age at transplant was 52.6 (12.6) years. Patients were mostly male (*n* = 113, 74.8%). Twenty-six patients (17.2%) were on durable MCS while 12 patients (7.9%) were on ECMO support at the time of transplantation. A total of 135 patients developed PGD, classified as follow: 31 patients (20.5%) with PGD-right ventricle and 104 patients (68.8%) with PGD-LV, distributed as 2 *mild*, 17 *moderate* and 85 *severe* PGD-LV.

**Table 1:** Patients’ characteristics (at time of transplantation)

Baseline characteristics of patients	All patients, n = 151	VIS_max_ ≤16th percentile, *n* = 25	VIS_max_ 17–50th percentile, *n* = 51	VIS_max_ 51–83rd percentile, *n* = 50	VIS_max_ ≥84th percentile, *n* = 25	*P*-Value
Recipient characteristics						
Age (years), median (IQR)	54 (47–62)	53 (44–65)	57 (53–62)	54.5 (48–63)	42 (36–58)	0.011
Gender (female), *n* (%)	38 (25.2)	8 (32)	14 (27.5)	13 (26)	3 (12)	0.37
** **Diabetes mellitus, *n* (%)	28 (18.5)	3 (12)	13 (25.5)	9 (18)	3 (12)	0.43
** **Body mass index (kg/m^2^)						
** **≤18.5	3 (2.0)	0 (0)	0 (0)	1 (2)	2 (8)	
** ** 18.5–25	81 (53.6)	14 (56)	26 (51)	27 (54)	14 (56)	0.18
** ** 25–30	48 (31.8)	8 (32)	22 (43.1)	12 (24)	6 (24)	
** ** >30	19 (12.6)	3 (12)	3 (5.9)	10 (20)	3 (12)	
Past history of cardiac surgery, *n* (%)	56 (37.1)	5 (20)	19 (37.3)	19 (38)	13 (52)	0.14
Aetiology of heart failure, *n* (%)						
** ** Dilated	69 (45.7)	14 (56)	20 (39.2)	19 (38)	18 (72)	
** ** Ischaemic	45 (29.8)	5 (20)	18 (35.3)	17 (34)	3 (12)	0.07
** ** Congenital	6 (4.0)	0 (0)	1 (2)	4 (8)	1 (4)	
** ** Others	31 (20.5)	6 (24)	12 (23.5)	10 (20)	3 (12)	
Long-term MCS, *n* (%)	26 (17.2)	3 (12)	6 (11.8)	10 (20)	7 (28)	0.29
Preoperative ECMO support, *n* (%)	12 (8)	2 (8)	5 (9.8)	5 (10)	0 (0)	0.44
Mechanical ventilation, *n* (%)	0 (0)	0 (0)	0 (0)	0 (0)	0 (0)	–
Creatinine clearance at transplant, *n* (%)						
** ** ≥60 ml/min/1.73 m^2^	94 (62.2)	19 (76)	31 (60.8)	27 (54)	17 (68)	
** ** ≥30 and <60 ml/min/1.73 m^2^	48 (31.8)	6 (24)	18 (35.3)	17 (34)	7 (28)	0.39
** ** <30 ml/min/1.73 m^2^ or dialysis	9 (6.0)	0 (0)	2 (3.9)	6 (12)	1 (4)	
Total bilirubin day of transplantation (mmol/l), median (IQR)	13 (8–22)	18 (8–24)	14 (7.5–26)	11 (8–18)	13 (9–19)	0.28
Donor characteristics						
Age (years), median (IQR)	52 (42–58)	52 (30–57)	49 (44–58.5)	52.5 (29.25–59.75)	52 (38–57)	0.80
Sex (female), *n* (%)	52 (34.4)	9 (36)	20 (39.2)	16 (32)	7 (28)	0.78
Tobacco abuse, *n* (%)	84 (55.6)	14 (56.0)	33 (64.7)	29 (58.0)	8 (32.0)	0.15
Diabetes mellitus, *n* (%)	12 (7.9)	2 (8.0)	3 (5.8)	5 (10.0)	2 (8.0)	0.43
Left ventricular ejection fraction, median (IQR)	60 (55–65)	61 (55–64)	65 (60–70)	60 (55–65)	60 (55–65)	0.20
End-diastolic interventricular septum (mm), median (IQR)	10 (9–12)	10 (8–12)	10 (9–12)	9 (8–11)	10 (9–11)	0.43
Cardiac arrest, *n* (%)	39 (25.8)	8 (32.0)	11 (21.6)	14 (28.0)	6 (24.0)	0.93
Norepinephrine at procurement (μg/kg/min), median (IQR)	0.18 (0.02–0.33)	0.06 (0.00–0.16)	0.18 (0.03–0.33)	0.23 (0.00–0.45)	0.21 (0.06–0.30)	0.10
ICU length (days), median (IQR)	3 (1–5)	3 (2–5)	3 (1–4)	4 (2–5)	3 (1–5)	0.78
Cause of death, *n* (%)						
** ** Cerebrovascular	69 (45.7)	10 (40)	29 (56.9)	18 (36)	12 (48)	0.19
** **Other	37 (24.3)	15 (60)	22 (43.1)	32 (64)	13 (52)	
Transplant characteristics						
Gender mismatch (female donor, male receiver), *n* (%)	27 (17.9)	4 (16)	10 (19.6)	8 (16)	5 (20)	0.96
Combined transplantation, *n* (%)	7 (4.6)	0 (0)	1 (2)	3 (6)	3 (12)	0.16
Ischaemic time (min), median (IQR)	198 (164.5–228.5)	199 (175–233)	192 (154.5–224.5)	197.5 (165–231)	200 (164–227)	0.95
Induction therapy (ATG), *n* (%)	148 (98.0)	25 (100)	50 (98)	48 (96)	25 (100)	0.66

ATG: antithymocyte globulin; ECMO: extracorporeal membrane oxygenation; IQR: interquartile range; MCS: mechanical circulatory support; SD: standard deviation; VIS_max_: vasoactive–inotropic score (calculated with maximum doses 24 h postoperatively).

#### *Vasoactive*–*inotropic score*

The median VIS_max_ 24 h after transplantation was 39.2 points (IQR = 19.4–83.0). Nine (6.0%), 15 (9.9%), 35 (23.2%), 20 (13.3%) and 72 (47.7%) had a VIS_max_ ≤5, between 5 and 15, between 15 and 30, between 30 and 45 and >45 points, respectively. For further analysis, we split the cohort according to the VIS_max_: ≤16th percentile (range = 0–15.2), 17–50th percentile (range = 15.3–39.2), 51–83rd percentile (range = 41.7–95.7) and ≥84th percentile (range ≥96.5 points). Supplementary Material, Table S1 summarizes the median dose of each vasoactive–inotropic drug administered in ICU during the first 24 h.

### Post-transplant outcomes

One year post-transplant survival was 82.8% (**Supplementary Material, Figure). The leading cause of death was multi-organ failure (*n* = 11, 42.3%) mostly related to septic shock in patients with persistently impaired graft function. Thirty-eight (25%) patients needed postoperative RRT for a median duration of 16 days [IQR = 7–28]. Forty-two patients (27.8%) presented septic shock, 56 (37%) developed VAP and 33 (21.8%) had a bloodstream infection. No patients underwent retransplantation during the study period.

### Primary end point—1-year mortality

In univariable analysis, among the 21 potential predictive variables analysed, 7 were associated with 1-year post-transplant mortality, including 3 recipients characteristics (age at transplant; gender; past history of cardiac surgery); 1 donor variable (age ≥50 years old); and 4 transplant characteristics (combined transplantation; VIS_max;_ category of VIS_max_; and category of PGD) (Table 2).

**Table 2:** Univariate analysis (Cox proportional hazard ratio)—all-cause mortality

Characteristics	Variable	Label	Patients (number)	Deaths (number)	HR	95% CI	*P*-Value
Recipient characteristics	Age (per 1-year increment)		151	26	1.046	[1.01–1.09]	0.014
	Gender	Male	113	15	1	–	
		Female	38	11	2.40	[1.10–5.22]	0.04
	Diabetes	No	123	20	1	–	
		Yes	28	6	1.34	[0.54–3.33]	0.54
	BMI (kg/m^2^)	<18.5	3	0	–		
		≥18.5 and <25	81	12	1	–	
		≥25 and <30	48	8	1.13	[0.46–2.75]	0.32
		≥30	19	6	2.22	[0.83–5.92]	
	Past history of	No	95	13	1	–	
	cardiac surgery	Yes	56	13	1.77	[0.82–3.82]	0.15
	Aetiology of heart	Dilated	69	10	1	–	
	Failure	Ischaemic	45	7	1.06	[0.40–2.79]	
		Congenital	6	2	2.86	[0.63–13.04]	0.55
		Others	31	7	1.59	[0.61–4.18]	
	Pre-HTx ECMO	No	139	26	1	–	–
		Yes	12	0	–		
	Long-term MCS	No	125	22	1	–	
		Yes	26	4	0.87	[0.30–2.52]	0.80
	Creatinine clearance (ml/min/1.73 m^2^)	≥60	94	13	1	–	
		≥30 and <60	48	10	1.58	[0.69–3.61]	0.27
		<30 or dialysis	9	3	2.72	[0.78–9.56]	
	Total bilirubin (per 1 mmol/l increment)		151	26	0.98	[0.94–1.02]	0.21
Donor characteristics	Age	<50 years	71	8	1	–	
		≥50 years	80	18	2.13	[0.93–4.90]	0.06
	Gender	Male	99	16	1	–	
		Female	52	10	1.20	[0.55–2.65]	0.65
	Cause of death	Cerebrovascular	69	14	1	–	
		Other	82	12	0.71	[0.33–1.53]	0.38
Transplant characteristics	Gender mismatch	No	124	21	1	–	
		Yes	27	5	1.09	[0.41–2.88]	0.87
	Weight mismatch	No	148	25	1	–	
		Yes	3	1	2.06	[0.28–15.23]	0.52
	Combined	No	144	22	1	–	
	Transplantation	Yes	7	4	1.90	[1.68–14.28]	0.01
	Cold ischaemia (per 1-h increment)		151	26	1.20	[0.76–1.89]	0.43
	Primary graft dysfunction	None	15	1	1	–	
		PGD-LV mild	2	0	–	–	0.05
		PGD-LV moderate	14	3	2.87	[0.30–27.6]	
		PGD-LV severe	65	20	4.09	[0.55–30.5]	
		PGD-RV	29	2	0.99	[0.09–10.9]	
	VIS_max_ (per 1-point increment)		151	26	1.004	[1.000–1.007]	0.07
	VIS_max_	≤16th percentile	25	1	1	–	
		17–50th percentile	51	6	3.11	[0.37–15.8]	0.03
		51–83rd percentile	50	11	6.02	[0.77–26.6]	
		≥84th percentile	25	8	9.03	[1.13–42.1]	

Weight mismatch was calculated as follows: (donor weight − recipient weight)/recipient weight × 100. Weight mismatch was considered when the result was lower than −30%.

BMI: body mass index; CI: confidence interval; HR: hazard ratio; MCS: mechanical circulatory support; PGD-LV: primary graft dysfunction-left ventricle; PGD-RV: primary graft dysfunction-right ventricle; Pre-HTx ECMO: pre-heart transplantation extracorporeal membrane oxygenation; VIS_max_: vasoactive–inotropic score (calculated with maximum doses 24 h postoperatively).

After multivariable analyses, VIS_max_ remained independently associated with 1-year mortality, even when forcing the variable PGD as defined by ISHLT guidelines (Table 3). We observed a stepwise increase in the risk of death with increasing VIS_max_: <16th percentile = reference, 17–50th percentile: hazard ratio (HR) = 2.61, 95% confidence interval (CI) = 0.30–15.8; 51–83rd percentile: HR = 4.08, 95% CI = 0.48–21.2; and ≥84th percentile: HR = 9.67, 95% CI = 1.12–37.1, *P* = 0.03. Other independent predictive variables included recipient age, recipient gender and combined transplantation (Table 3).

**Table 3:** Multivariable analysis

Variables	Label	Patients (number)	Deaths (number)	HR	95% CI	LRT, *P*-value
Recipient age	Per 1-year increment	151	26	1.046	[1.001–1.084]	0.013
Recipient gender	Male	113	15	1	–	0.047
	Female	38	11	2.23	[1.01–5.08]	
Combined	No	144	22	1	–	0.048
transplantation	Yes	7	4	2.85	[1.01–9.58]	
Primary graft dysfunction	None	15	1	1	–	
	PGD-LV mild	2	0	–	–	
	PGD-LV moderate	14	3	1.44	[0.14–15.1]	0.47
	PGD-LV severe	65	20	1.45	[0.18–12.0]	
	PGD-RV	29	2	0.45	[0.04–5.20]	
VIS_max_	≤16th percentile	25	1	1	–	0.03
	17–50th percentile	51	6	2.61	[0.30–15.8]	
	51–83rd percentile	50	11	4.08	[0.48–21.2]	
	≥84th percentile	25	8	9.67	[1.12–37.1]	

CI: confidence interval; HR: hazard ration; ICU: intensive care unit; LOS: length of stay; LRT: likelihood-ratio test; PGD-LV: primary graft dysfunction-left ventricle; PGD-RV: primary graft dysfunction-right ventricle; VIS_max_: vasoactive–inotropic score (calculated with maximum doses 24 h postoperatively).

### Risk stratification

We analysed the improvement in stratification of the risk of death during the first-year post-transplant when adding VIS_max_ on top of the current ISHLT PGD classification. The discrimination of PGD classification alone, as assessed by the *c*-statistic, was 0.64 (95% CI = 0.55–0.73). The addition of VIS_max_ significantly improved discrimination (*c*-statistic = 0.72, 95% CI = 0.62–0.82, *P* = 0.06). Reclassification indices were also significantly improved. The non-event NRI was 0.227 and the event NRI was 0.243. Global NRI was 0.47 representing a significant improvement in reclassification (*P* = 0.03). Similarly, the analysis of IDI revealed a significant improvement in reclassification (IDI = 0.043, *P* = 0.04).

### *Secondary* end points*: early morbidity*

**Figure 1:**
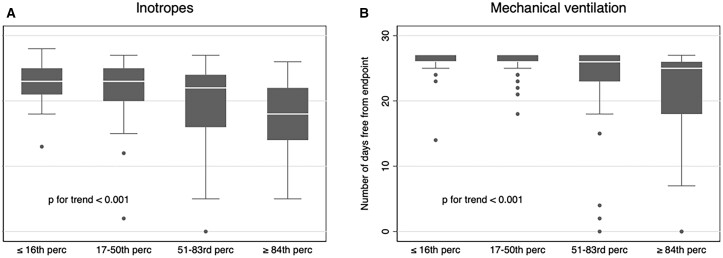
(**A**) Number of days alive free from inotrope infusion during the first 30 days post-transplantation, according to VIS_max_. (**B**) Number of days alive free from mechanical ventilation during the first 30 days post-transplantation, according to VIS_max_. VIS: vasoactive–inotropic score.

We found a significant association between VIS_max_ and various post-transplant events during the first 30 days post-transplantation, including number of days alive free from infused inotropes (*P*-value < 0.001; Fig. 1A), number of days alive free from mechanical ventilation (*P*-value < 0.001; Fig. 1B), ICU LOS (*P*-value = 0.002), occurrence of bloodstream infections, septic shocks, VAP and RRT (*P*-value < 0.001 for all comparisons).

### Internal validation

Among 200 bootstrap samples randomly generated for internal validation procedures, VIS was independently associated with 1-year all-cause mortality in 72% of the samples. VIS was the most selected variable, ahead of recipient age (68% of samples) and recipient sex (58% of samples). These results remained stable when forcing the variable ‘PGD’ into the automatically generated multivariable models.

## DISCUSSION

In this study conducted in a contemporary cohort of 151 heart transplant recipients, VIS_max_ calculated in the first 24 h after ICU admission was independently associated with morbidity and 1-year mortality and significantly improved risk stratification of early death on top of the current ISHLT PGD classification.

When it comes to adult HTx, most studies concentrated on scores based on preoperative factors. Barge-Caballero *et al.* [15] calculated preoperative VIS and noted a continuous association between preoperative VIS and post-transplant mortality. Only 1 retrospective study by Venema *et al.* [18], involving 81-transplanted patients, calculated postoperative inotrope score and reported that it was significantly associated with 5-year survival. In line with their results, VIS_max_ independently predicted 1-year mortality in our work albeit some discrepancies between the studies. First, we found that PGD was common after HTx and frequently required ECMO. Whether inotrope score remained discriminant in a population under VA-ECMO was unclear so far. Venema *et al.* [18] reported a significant interaction between VIS_max_ and MCS but the study was underpowered to assess this subgroup. Our data suggest VIS_max_ still accurately predict outcomes for severe PGD requiring MCS. The high rate of VA-ECMO after HTx in our centre might be explained by local protocol and is supported by recently published data stating that VA-ECMO for HTx does not burden survival but could prevent from acute cardiovascular collapse and multiple organ failure [17]. Second, we used VIS_max_ calculated during 24 h after HTx (versus mean inotrope score calculated during first 48 h for Venema *et al.*), which prevents from effective between-studies comparison. We considered VIS_max_ as it could be easily translated into clinical practice and can provide early prognostication.

In a previous study involving >3000 patients, the predictive accuracy of VIS_max_ was evaluated for a composite outcome, which included 30-day mortality, mediastinitis, stroke, acute kidney injury and myocardial infarction in conventional cardiac surgery [13]. They reported VIS_max_ predicted a composite of unfavorable outcomes and mortality up to 1-year after surgery. Median VIS_max_ was 4.0 (IQR = 0.0–14.6), contrasting with our results. Heart transplant was associated with elevated VIS_max_ mainly derived by PGD in our cohort.

**Figure 2:**
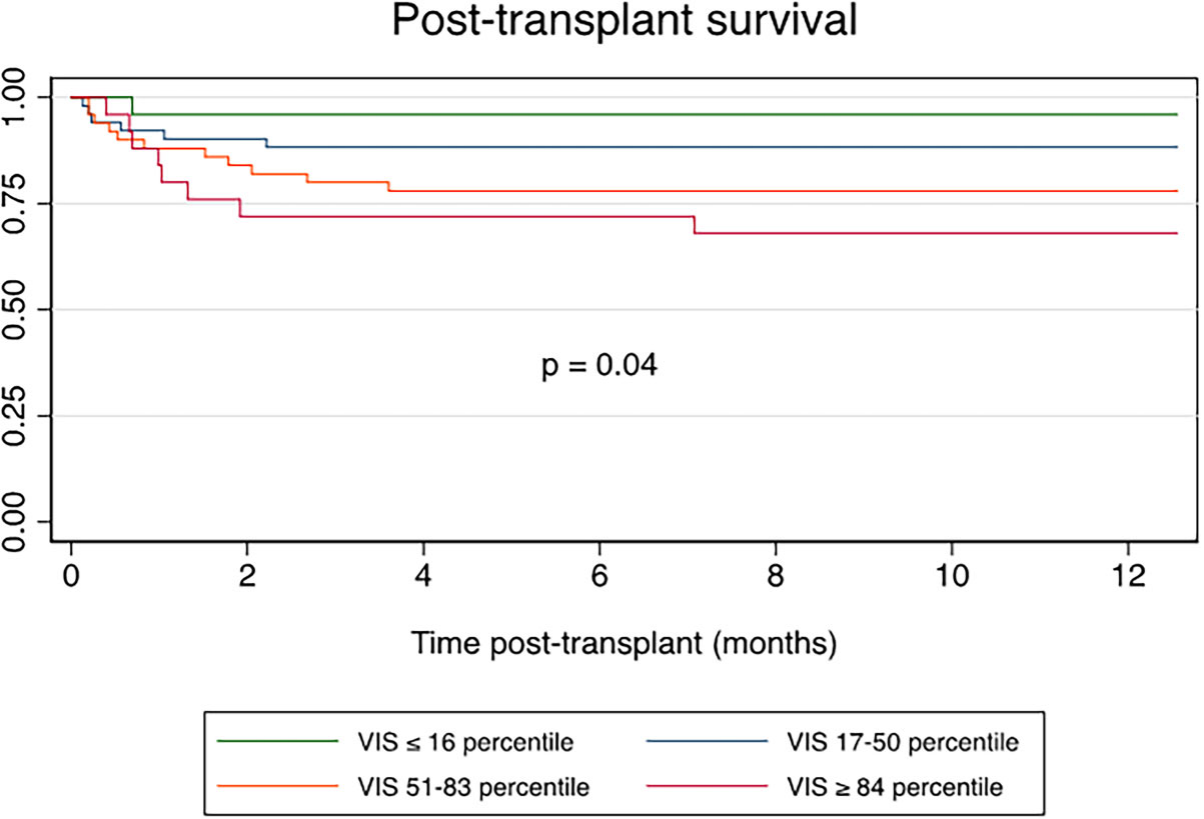
Overall post-transplant survival at 1 year according to VIS_max_ category. VIS: vasoactive–inotropic score.

It is valuable to notice that survival curves (Fig. 2) split between 0 and 3 months but remained parallel afterward, suggesting VIS_max_ rather correlates with mid-term outcome. Initial exposure to inotrope or vasopressors has no predictive value for long-term survival once patients discharged alive from hospital. In the literature, VIS was also an independent predictor of in-hospital mortality after left ventricular assist device when calculated after stabilization in the operating room and upon arrival to ICU [19]. In paediatric HTx, death tended to be higher in those with persistent high VIS until 48 h postoperatively, although not statistically significant [14]. Altogether, VIS_max_ could be considered as a relevant stratification score for comparing transplanted patients.

In addition, this study showed that adding VIS_max_ to the current ISHLT PGD classification significantly improved risk stratification after HTx as illustrated by the global net reclassification index of 0.47. However, discrimination of the model remained limited and not clinical relevant for individual risk stratification. In their study, Tadros *et al.* [20] noted that for VIS, the ROC curve exhibits 10 as the ideal cut-off with area under the curve >0.8 for PGD.

Our study also showed, in accordance with other studies, that higher VIS_max_ was significantly associated to number of days free from mechanical ventilation and infused inotropes in ICU, and ICU LOS [14, 16, 19]. Moreover, risk for bloodstream infections, septic shock and VAP appears constant among VIS_max_ percentiles. In fact, McIntosh *et al.* [21] showed that, in paediatric sepsis, VIS was independently associated with ICU LOS, ventilator days, and cardiac arrest/ECMO/mortality. The increased risk of infection along with increasing VIS_max_ could be explained by the frequent use of mechanical, respiratory, and circulatory supports in severe patients, who were prevalent in our study, as well as by immune dysfunction secondary to circulatory failure and/or excess of inotropes. Pons *et al.* [22] have shown that in patients who had HTx, postoperative ECMO was an independent risk factor for early non-viral infection. Finally, VIS_max_ increments were linearly associated with increased HR for RRT requirement after HTx as was also demonstrated by other studies [18, 23]. Hou *et al.* [24] showed that VIS_max_ was an independent predictor of postoperative acute kidney injury in adult patients after cardiovascular surgery and increased prognostic accuracy of Society of Thoracic Surgeons score, allowing a risk reclassification. Hence using VIS_max_ as an indicator for renal aggression could help implement early nephroprotective measures.

### Limitations

This study has 4 main limitations. First, the small sample size with limited number of ‘events’ such as death reduced the power of statistical analysis. It was reflected in the wide confidence interval of OR in the cox model. Second, this is a single-centre retrospective study. Despite being retrospective, there were few missing data, but it lacks an external validation cohort. Third, 1 maximum value of inotropic and vasoactive support may be subjected to confounding factors such as momentary deeper sedation, postoperative bleeding/hypovolaemia, or procedures requiring temporarily increased pharmacological cardiovascular support. Albeit VIS_max_ appears easier for clinical practice, whether total cumulative inotrope and vasoactive exposure during the first 24 h in ICU could better predict outcomes remains unknown. Finally, the population mainly composed of severe PGD, might burdened external validity of the study. The high prevalence of severe PGD in French cohorts compared to literature might be explained by donors’ higher mean age (48 ± 14.4 years old), previously published as a major risk factor for PGD [25]. It is valuable to highlight those results might have been influenced by local practice and centre experience as our hospital is referred for VA-ECMO and trained medical, surgical and paramedical teams significantly reduce VA-ECMO related complications [26, 27]. However, exposure to VA-ECMO did not change the interpretation of the results and VIS_max_ remained reliable even among severe PGD patients.

## CONCLUSION

To our knowledge, this is the first study describing adverse outcomes and mortality after adult HTx based on post-transplant VIS_max_. It confirms that VIS_max_ calculated during the first 24 h after postoperative ICU admission is independently associated with 1-year mortality. In addition, ICU LOS, duration of mechanical ventilation and infused inotropes increased with increasing VIS_max_. Future prospective multi-institutional studies are necessary to better evaluate the utility of postoperative VIS_max_ after HTx.

##  

Presented at the French Society of Anesthesia & Intensive Care Medicine (SFAR) Congress, Paris, France, 24 September 2021.

## SUPPLEMENTARY MATERIAL

Supplementary material is available at *ICVTS* online.

## Funding

None.

**Conflict of interest:** none declared.

## Supplementary Material

ivad055_Supplementary_Data

## Data Availability

If requested, we shall produce the data upon which the article is based for examination by the editors or their assignees. **Joanna Tohme:** Conceptualization; Data curation; Formal analysis; Investigation; Methodology; Project administration; Writing—original draft. **Mickael Lescroart:** Formal analysis; Investigation; Writing—original draft. **Jérémie Guillemin:** Data curation; Investigation; Writing—original draft. **Pascal Orer:** Data curation; Investigation. **Pauline Dureau:** Validation; Writing—review & editing. **Shaida Varnous:** Validation; Writing—review & editing. **Pascal Leprince:** Validation; Writing—review & editing. **Guillaume Coutance:** Conceptualization; Data curation; Formal analysis; Investigation; Methodology; Project administration; Supervision; Validation; Visualization; Writing—review & editing. **Adrien Bouglé:** Conceptualization; Formal analysis; Investigation; Methodology; Project administration; Supervision; Validation; Visualization; Writing—review & editing. Interdisciplinary CardioVascular and Thoracic Surgery thanks Christoph Knosalla, Stephen Pettit, Stefano Mastrobuoni and the other anonymous reviewer(s) for their contribution to the peer review process of this article.

## ABBREVIATIONS

CI: Confidence interval
HR: Hazard ratio
HTx: Heart transplantation
IDI: Integrated discrimination improvement
IQR: Interquartile range
MCS: Mechanical circulatory support
NRI: Net reclassification improvement
PGD: Primary graft dysfunction
PGD-LV: PGD-left ventricle
RRT: Renal replacement therapy
VAP: Ventilator-associated pneumonia
VIS: Vasoactive–inotropic score
