# Supplementary material for: Association between vasoactive–inotropic score, morbidity and mortality after heart transplantation
Source: Interdiscip Cardiovasc Thorac Surg. 2023 Apr 17;36(4):ivad055. doi: 10.1093/icvts/ivad055 (PMC10118996; doi:10.1093/icvts/ivad055)
Supplement: ivad055_Supplementary_Data [file ivad055_supplementary_data.zip › Supplementary Table 1.docx]

**Supplementary Table 1. Median dose of vasoactive-inotropic drugs during the first 24 hours after intensive care unit admission**

|  | **Epinephrine (µg/Kg/min)** | **Norepinephrine (µg/Kg/min)** | **Dobutamine (µg/Kg/min)** |
| --- | --- | --- | --- |
| **Median dose [IQR]** | 0.35 [0.23-0.56] | 0.38 [0.16-0.68] | 10 [6-10] |
