# Supplementary material for: Association between vasoactive–inotropic score, morbidity and mortality after heart transplantation
Source: Interdiscip Cardiovasc Thorac Surg. 2023 Apr 17;36(4):ivad055. doi: 10.1093/icvts/ivad055 (PMC10118996; doi:10.1093/icvts/ivad055)
Supplement: ivad055_Supplementary_Data [file ivad055_supplementary_data.zip › Supplementary Figure_R1.docx]

**Supplementary Figure. Overall post-transplant survival at one year.**
